# First isolation of *Dicrocoelium dendriticum* among cattle in some Northern Algerian slaughterhouses

**DOI:** 10.14202/vetworld.2019.1039-1045

**Published:** 2019-07-16

**Authors:** Linda Chougar, Kh. Harhoura, M. Aissi

**Affiliations:** Animal Health and Productions Laboratory, High National Veterinary School, B.P.228, Oued Samar, Algiers, Algeria

**Keywords:** bile, cattle, dicrocoeliasis, *Dicrocoelium dendriticum*, liver, stools

## Abstract

**Background and Aim:**

*Dicrocoelium dendriticum* or small liver fluke often causes unnoticed clinical manifestations in cattle. For a live animal, its diagnosis is mainly based on the detection of eggs by coproscopic examination. The objective of this study was to determine the presence or absence of *Dicrocoelium* spp. never previously reported in the study area but also to establish its prevalence, as well as an association between dicrocoeliasis and sex, age and season of the year, and histological characteristics.

**Materials and Methods:**

The study was carried out in slaughterhouses of three districts (Bouira, Tizi-Ouzou, and Bejaia) from January 2017 to December 2017. To this end, of 4053 cattle, representing more than 10% of the total number of animals slaughtered, stool and bile samples were collected and a liver inspection was carried out to investigate lesions of distomial cholangitis. They were processed for histological analysis. The specimens were morphologically identified according to the orientation of the testicles, the length and width of the body, and the level of the maximum width of the body.

**Results:**

The total prevalence of dicrocoeliasis obtained of the 4053 cattle inspected is 0.52% with a prevalence of 0.66% in Tizi-Ouzou, 0.54% in Bouira, and 0.27% in Bejaia. About 0.52% of livers had distomial cholangitis (21 of the 4053 livers examined had adult *D. dendriticum* and 15% had non-distomial cholangitis. About 0.25% of cattle had *D. dendriticum* eggs in the stool versus 0.52% of cattle had parasite eggs in the bile. Statistical analysis revealed no significant association between dicrocoeliasis infection and the season of the year (p>0.05). However, a significant association was found between dicrocoeliasis infection and sex and age of the animal (p<0.05); females and older animals are more likely to have dicrocoeliasis. Histological analysis of the fluke revealed an anterior positioning of the testicles with a slightly oblique tandem orientation, an average body length of 3.69 mm and an average body width of 1 mm. The maximum body width level is either in the middle of the fluke body or in the rear position.

**Conclusion:**

The histological study confirms that the collected fluke is *D. dendriticum*. Thus, this work reveals for the 1^st^ time in Algeria the presence of *D. dendriticum* in three districts (Bouira, Tizi-Ouzou, and Bejaia). The results indicate that many cattle farms in the North Central Province of Algeria are infested with *D. dendriticum*.

## Introduction

*Dicrocoelium dendriticum*, which causes liver fluke disease in ruminants and is of zoonotic and economic importance. It is prevalent in many regions of the world [1-3]. Although it has been identified in America, Asia, North Africa, and Europe, dicrocoeliasis is a little-known parasitic disease [4]. *D. dendriticum* lives in the adult stage, in the bile ducts, canaliculus, and gallbladder of its hosts (cow, sheep, goat, and pig) [5-7]. Humans can become accidentally infected by swallowing ants on vegetation or on various fruits. Children are, therefore, affected more frequently [8]. From an economic point of view, *D. dendriticum* causes a lot of damage to the livestock industry every year [9].

This parasite, which lives in the liver of its definitive host, involves a gastropod snail as the first intermediate host and an ant as the second intermediate host in its life cycle. Sporocysts and cercariae, which are larval stages of the parasite, live in the hepatopancreas of terrestrial snails, metacercariae are also larval stages, in the abdomen and brain of ants (second intermediate host). The parasite has a slight specificity of the definitive host, with a preference for ruminants, which can be considered as the true host of origin, including mainly sheep and goats, and secondarily large ruminants such as cattle [10]. The number of reports on dicrocoeliasis is increasing in many countries due to the expansion of dry habitats and parasites that have become resistant to antihelminthics [11].

In Algeria, only one study reported the presence of dicrocoeliasis in the Mitidja’s area (Northern Algeria) with a prevalence in cattle of 0.07% and 0.86% corresponding to fluke and eggs positive cases [12]. However, no data on dicrocoeliasis have been reported in Tizi-Ouzou, Bouira, and Béjaia (Kabylie region).

This study aimed to investigate the presence and prevalence of dicrocoeliasis among cattle with histological characteristics in several slaughterhouses from three districts in Northern Algeria and to detect factors associated with *D. dendriticum* infection.

## Materials and Methods

### Ethical approval

The study was not conducted on live animals, but only on animals slaughtered at approved slaughterhouses as a routine slaughtering practice.

### Study area

**Figure-1:**
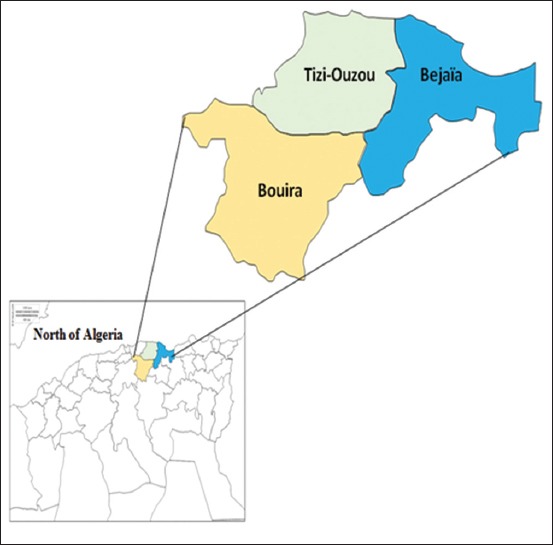
Map of the study area (Bouira, Tizi-Ouzou, and Béjaia). [Source: https://d-maps.com/index.php?lang=fr].

The studies were carried out in several slaughterhouses from three districts in Northern Algeria: Tizi-Ouzou, Bouira, and Bejaia (Kabylie region). This area is characterized by the Mediterranean climate and is divided by two geographical barriers, the Tell and the Sahara Atlas. Northern Algeria is characterized by a cold winter and a warm summer with heavy rainfall (Figure-1).

### Identification of the animals

Samples were collected daily from January 2017 to December 2017. The selection of slaughterhouses per day was randomly chosen. An investigation report form was completed for each sampled cattle, indicating the origin of the animal, which was determined from information provided by the farmers, the sampling season, sex, and age of the animal, which was determined by dentition and confirmed by the slaughter orientation form signed by the veterinarian. All sampled cattle were categorized into three age groups: <2 years, 2-4 years, and >4 years and finally the appearance of liver during the inspection.

### Collected samples

#### Stool sample

After evisceration of the carcass, 150 g of feces samples were collected from the rectum and placed in a box, then stored in the refrigerator at +4°C until analyzed.

#### Bile sample

Bile was collected by puncturing the gallbladder with a 5 cc syringe. The bile thus collected was stored in the refrigerator at +4°C until it was analyzed.

#### Flukes sample

The specimens of *Dicrocoelium* were collected after necrotic inspection of the livers of slaughtered cattle at various slaughterhouses in the North Central Province of Algeria. The specimens were morphologically identified based on the descriptions made by Yamaguti [13], Hinaidy [14], Taira *et al*. [15], Otranto *et al*. [16], and Bourgat *et al*. [17].

### Diagnostic methods

#### Liver inspection at the slaughterhouse

The inspection of the livers took place after total evisceration and cutting of the carcass. It was carried out through two mandatory regulatory incisions. The first was broad and superficial located at the level of the large bile ducts at the base of the pallet and the second was short and deep perpendicularly located at the level of the caudate lobe.

#### Stool analysis (coprological analysis)

Coprological analysis was performed by two methods, namely, the sedimentation technique using the Dinnik and Dinnik sedimentation method [18] and the flotation technique based on the Teuscher method [19].

#### Bile analysis

The bile contained in the syringes was transferred into conical tubes. The tubes were centrifuged (at 5000 rpm for 1 min) for 10 min. About 2 ml of the biliary pellet obtained were sucked up with a Pasteur pipette and placed on a slide and covered with a slide. The sample was observed under an optical microscope at 100× and 400× to determine the presence of *D. dendriticum* eggs.

#### Flukes preparation for histology

After collecting the parasites, the flukes were washed with phosphate-buffered saline to remove all traces of bile and were fixed at 10% formalin for at least 24 h by immersing them in 10 times their volume. At the end of this fixation, the histological treatment and coating of the paraffin were carried out using conventional techniques. The sections from 3-4 μm were obtained by microtome of each block and stained with hematoxylin and eosin according to standard histological protocols. The sections were examined and measured with a micrometer.

### Statistical analysis

Data analysis was performed using IBM SPSS Statistics 22.0 software. Tests used the Pearson Chi-square test. A significant association between animal positivity to dicrocoeliasis and the sex, age, and season of the year was determined if p<0.05.

## Results

**Figure-2:**
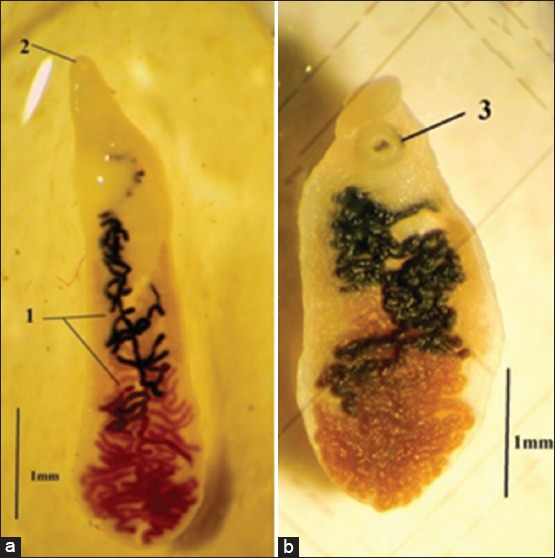
Adult *Dicrocoelium dendriticum* observed under a magnifying glass: (a) Adult parasite elongated form; (b) Adult parasite squat form (2: Oral sucker, 1: Uterus, and 3: Ventral sucker) with a translucent body.

**Figure-3:**
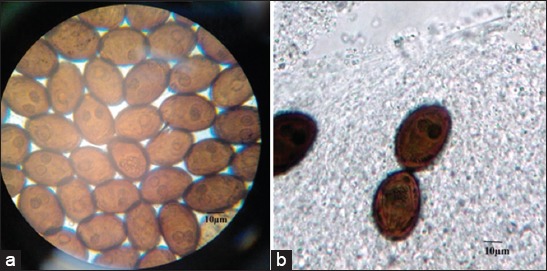
Numerous eggs of *Dicrocoelium dendriticum* in bile and stools observed under an optical microscope (Gr. 1000×). (a): Parasite eggs in the bile, (b): Parasite eggs in the stool (the egg is globally ellipsoid (asymmetrical), dark brown in color and capped).

The results showed that the total prevalence of dicrocoeliasis was 0.52%, which represents 21 positive cattle of 4053. The parasitized livers presented lesions (Table-1), parasite (Figure-2a and b), and parasite eggs in bile (0.52%) (Figure-3a). However, only 10 (0.25%) of affected cattle revealed the presence of *D. dendriticum* eggs in coprology (Figure-3b and Table-1). The study included 1658 cattle from Tizi-Ouzou region. Among them, 11 (0.66%) were found positive for dicrocoeliasis with the presence of the parasite and eggs. In Bouira Province, of 1304 cattle inspected and analyzed, only 7 cattle (0.54%) were positive for dicrocoeliasis with the presence of the parasite and eggs.

**Table 1:** Summary of different diagnostic techniques in the three districts (Bouira, Tizi-Ouzou, and Béjaia).

Pathological	Wilayas			

	Bejaia n (%)	Tizi-Ouzou n (%)	Bouira n (%)	Total n (%)
Bile analysis				
+^c^	3 (0.27)	11 (0.66)	7 (0.54)	21 (0.52)
−^d^	1088 (99.7)	1647 (99.3)	1297 (99.4)	4032 (99.48)
Coproscopic analysis				
+^c^	2 (0.18)	5 (0.3)	3 (0.23)	10 (0.25)
−^d^	1089 (99.8)	1653 (99.7)	1301 (99.7)	4043 (99.75)
Liver inspection				
Chol+dist+^a^	3 (0.27)	11 (0.66)	7 (0.54)	21 (0.52)
Chol+dist−^b^	167 (15.3)	312 (18.8)	130 (9.96)	609 (15)
Healthy	921 (84.4)	1335 (80.5)	1167 (89.5)	3423 (84.46)

a Chol+dist+=Distomian cholangitis (inflammation of the bile ducts with the presence of the parasite),

b Chol+dist−=Non-distomian cholangitis (inflammation of the bile ducts without the presence of the parasite),

c (+)=Presence of eggs of *Dicrocoelium dendriticum*,

d (−)=Absence of eggs of *D.dendriticum*

Whereas Bejaia Province has the lowest prevalence among the 1091 cattle inspected and analyzed; only 3 cattle (0.27%) were positive for dicrocoeliasis with the presence of eggs and adults of the parasite (Table-1).

### Association between dicrocoeliasis and sex, age, and season

The risk factors (age, sex, and season) are the same for the three districts (Tizi-Ouzou, Bouira, and Bejaia); therefore, the entire cattle herd in the three districts was selected for the statistical study.

#### Season

Among 0.52% of positive cases, 0.15% were identified in summer, 0.12% in autumn, and 0.22% in spring, compared to a prevalence of 0.02% in winter. No significant association between the positivity of *D. dendriticum* and season was observed (p>0.05) (Table-2).

**Table 2:** Summary of positive cases according to risk factors (seasons, sex, and age).

Risk factors	Infection	

	+ (%)	− (%)
Seasons		
Autumn	5 (0.12)	1034 (25.5)
Winter	1 (0.025)	1003 (24.74)
Spring	9 (0.22)	1016 (25.1)
Summer	6 (0.15)	979 (24.15)
Sex		
Male	6 (0.15)	3534 (87.2)
Female	15 (0.37)	498 (12.3)
Age (years)		
Aged (>4)	10 (0.25)	319 (7.87)
Intermediate (2-4)	9 (0.22)	1090 (26.9)
Young (<2)	2 (0.05)	2623 (64.7)

#### Age

Of the 0.52% of positive cases, 0.25% were aged (>4 years), 0.22% were middle-aged (2-4 years), and 0.05% were young (<2 years). There is a significant association between the positivity of the animal to *D. dendriticum* and the age class (p<0.05) (Table-2).

#### Sex

Of the 0.52% of positive cases, 0.37% were female and 0.15% were male. There is a significant difference according to the animal’s sex (male or female) for *D. dendriticum* (p<0.05) (Table-2).

### Morphological measurements

**Figure-4:**
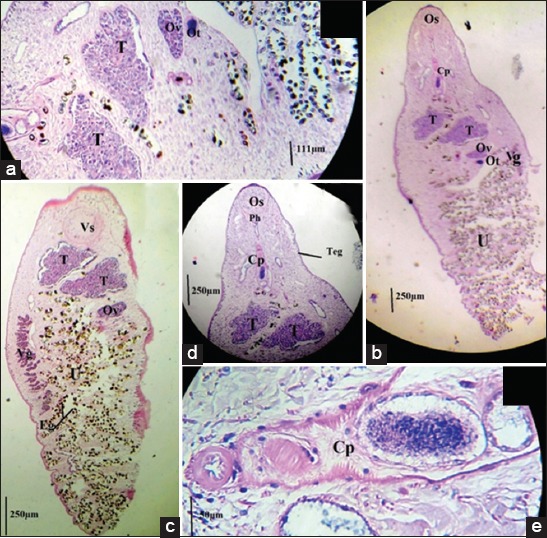
Histological section of an adult of *Dicrocoelium* *dendriticum*: (a) Histological section of the middle part of the parasite; (b) Complete histological section of the parasite “dorsal face;” (c) Complete histological section of the parasite “ventral face;” (d) Histological section of the anterior parasite; (e) Histological section of the cirrus pouch. (Cp: Cirrus pouch, Os: Oral sucker, Vs: Ventral sucker, T: Testes, Ov: Ovary, Ot: Ootype, Ph: Pharynx, U: Uterus filled with brown eggs of parasites (Eg), Teg: Its tegument is smooth).

Table-3 presents the results of the morphological measurements. Flukes’ body was flattened, translucent lanceolate, narrower in the anterior region than in the posterior region. The average body length was 3.69 mm and the maximum average body width was 1 mm at the vitelline glands (Vg) or in the posterior part of the body. Its integument is smooth (Teg) (Figure-4d).

**Table 3:** Morphometric data of *Dicrocoelium dendriticum* (from cattle).

Body/ organs	Items	*Dicrocoelium dendriticum* (μm)	

		Minimum-Maximum	Mean
Body	Length	2600-4600	3690
	Width	1000-1200	1000
Oral sucker	Diameter internal	90-111	103.66
	External	245-285	271.76
Ventral sucker	Diameter internal	102-120	113.19
	External	312-366	344
Testes	Length	250-300	272.14
	Width	480-580	537.42
Ovary	Length	90-115	107
	Width	180-285	230
Vitelline glands	Length	855-1500	1107.71
Eggs in the uterus	Length	31.6-38.8	34.19
	Width	20.8-25.6	22.44
Cirrus pouch	Length	420-450	439
	Width	120-140	135.04
Ootype	Length	60.2-67.9	65.5
	Width	102-111	105.9

The oral sucker (Os) was round and located at the anterior end of the body (Figure-4b). The mean external diameter of the buccal sucker was 271 μm. Under the buccal sucker, a small round pharynx (Ph) (60 μm) followed by a fine esophagus was observed (Figure-4d). The ventral sucker (Vs) was round and the mean external diameter was 344 μm (Figure-4c).

The two globular testicles (T) were located immediately after the Vs, are arranged in slightly oblique tandem orientation so that their size varied from 250 to 300 µm for length and 480 to 580µm for width (Figure-4a-d). The posture of the cirrus pouch (Cp) was voluminous and elongated axial (420-450 µm) (Figure-4e). Figure-4 also shows that the smallest ovary (Ov) was located directly behind the testicle and that the ootype (Ot), measuring 60.2-67.9 µm long, was located at the bottom of the ovary (Figure-4a).

The Vg (855-1500 µm) occupied 1/3 of the parasite body in a central position and did not reach the posterior edge of the ovary (Figure-4c). The uterus (U) occupied the posterior 2/3 of *Dicrocoelium* spp. body, characterized by numerous convolutions (Figure-4c).

The eggs (Eg) were ellipsoidal, slightly asymmetric, measuring 31.6-38.8 µm long and 20.8-25.6 µm wide. They occupied the entire part of the uterus with a very light brown color in the posterior part of the parasite. In the anterior part, they were very dark brown and mature with a small operculum on one pole and surrounding two lighter vesicles on the opposite pole (Figure-4c).

## Discussion

During this study, *D. dendriticum* was found for the 1^st^ time in some slaughterhouses in the Northern Province of Algeria, namely Bejaia, Tizi-Ouzou, and Bouira with a prevalence of 0.27%, 0.66%, and 0.54%, respectively, for a total prevalence of 0.52%. The results are consistent with those obtained by many authors with dicrocoeliasis prevalences increasing from 1.47, 1.76, and 2.10% in 1999-2000 to 0.69, 0.34, and 0.25% in 2003-2004 in cattle, sheep, and goats successively [20].

In addition, 1, 0.8, and 1% of livers seized due to *D. dendriticum* in sheep, goats, and cattle, respectively, were reported in Western Iran [21]. In Arak, Iran, Arbabi *et al*. [22] indicated a prevalence of 0.77% in cattle, goats, and sheep. While in the Mitidja region of Algeria, a prevalence of 0.07% and 0.86% in cattle was recorded, corresponding, respectively, to flukes and eggs positive cases [12].

Other authors have noted higher prevalences, notably Schweizer *et al*. [23], with 47.2% of cases of dicrocoeliasis in Swiss farm cattle; Jithendran and Bhat [24] found 8.1% of sheep and 4.1% of goats positive for dicrocoeliasis in India; in Northern Niger, the prevalence of dicrocoeliasis was 56% in cattle, 13.1% for sheep, and 5.2% for goats [25]. Asanji and Williams [26] reported that 61.8% of cases of in cattle in Sierra Leone; Aminzare *et al*. [27] found that 5.95% of livers of sheep and goats slaughtered at the Nishapour slaughterhouse were condemned.

Coproscopic analysis detected the presence of *D. dendriticum* eggs in only 10 cattle, a prevalence of 0.25%. These results are comparable to those obtained by Levasseur [28] who showed a low fecal excretion of *Dicrocoelium* eggs in cattle, with a number of PGOs ranging from 0 to 250, and an average close to 15, which very often makes the diagnosis false negative.

Other authors reported higher prevalences. In Italy, Cringoli *et al*. [29], according to a coprological study of 81 cattle and 197 sheep farms, reported that 53.1% of cattle farms (43 of 81 farms) were positive for *Dicrocoelium* (16% of animals) and 133 of 197 sheep farms (67.5%) were positive for *D*. *dendriticum*. Bihaqi *et al*. [30] also noted a prevalence of 3.44% of *Dicrocoelium* spp. in goats in India based on a coprological study.

### Association between dicrocoeliasis and sex, age, and season

The statistical analysis revealed no significant association between dicrocoeliasis and season (p>0.05), with a higher prevalence in spring (0.22%), followed by summer (0.15%), autumn (0.12%), and finally winter (0.025%).

The results are comparable to those obtained by Arbabi *et al*. [22] who reported no significant association between dicrocoeliasis and the season of the year. However, other authors have reported results with a significant association between season and dicrocoeliasis in sheep, cattle, and goats in Western Iran (p<0.001). Hepatic condemnations due to dicrocoeliasis were frequent in autumn for sheep and cattle and in winter for goats [21].

This difference with the results of the present study may be caused by different environmental conditions. Iran is generally an arid country, but the west and north are rainier than the east and south. While Northern Algeria has a Mediterranean climate, summers are hot and dry and winters are warm and rainy and sometimes snowy.

On the other hand, the results also showed a significant association between dicrocoeliasis and sex, age of animal (p<0.05); of 513 (12.6%) females, 15 (0.37%) were positive and of 3540 (87.3%) males, only 6 (0.15%) were positive, so females seem much more vulnerable to this disease.

This could be due, on the one hand, to their longer lifespan than males, which makes them more exposed to meadow and disease development, their physiology (gestation and lactation) leading to a reduction in immunity and more sensitive to parasitic diseases.

On the other hand, the female breeding methods used by farmers in Tizi-Ouzou, Bouira, and Bejaia Provinces are often extensive. Females tend to graze and therefore to be more exposed and infested, unlike males, whose breeding is often intensive because they are intended for fattening. In addition, the nature of their diet often consists of cereals in seed or ground, which exposes them to a lower risk of infestation.

With regard to the age factor, there seems to be a direct link with the sex factor, as males are slaughtered at a younger age, unlike females whose slaughter before 5 years is prohibited, except in the case of emergency and stamping out. The results obtained are comparable to those of Bihaqi *et al*. [30] who reported a higher incidence of gastrointestinal helminths among females than among males.

Dicrocoeliasis affecting cattle causes economic losses due to decreased milk production, reduced carcass weight, possible sterility, infertility, and finally seizure of parasitized livers at the slaughterhouse [8].

Histological analysis of *Dicrocoelium* shows that the flukes collected in Algeria are *D. dendriticum* of the anterior positioning of the testicles in tandem or slightly oblique unlike *D. chinensis*, which are in bilateral position [16], and the maximum width of the body is in the middle or in the posture of the body.

The parasite body is flat and translucent revealing all organs. The results show shorter parasite sizes ranging from 2.8 to 4.5 mm, which differs from the more elongated *Dicrocoelium hospes* [17].

## Conclusion

This work aimed to determine the presence or absence of *D. dendriticum* never reported before in Algeria. The results revealed the presence of *D. dendriticum* in three districts: Tizi-Ouzou, Bejaia, and Bouira. Its low prevalence confirms the beginning of the introduction of the parasite in Algeria, which could increase in the coming years if no measures are taken.

The province of Tizi-Ouzou has the highest rate of infested cattle followed by Bouira and finally Bejaia. In addition, the results show that females and older animals are more likely to be affected by dicrocoeliasis. Animals with several years of grazing are more likely to excrete *D. dendriticum* eggs than those in the 1^st^ year of grazing.

Many studies on dicrocoeliasis among different bovine, sheep, and goat species must be carried out in Algeria both on its prevalence and its epidemiology. In particular, molecular identification of the small fluke would be useful to determine the presence of intraspecific variation of the species present in Algeria.

## Authors’ Contributions

LC and KH carried out the inspection of cattle livers in the different slaughterhouses, LC collected the samples (stools and bile) and performed the experiments. LC and MA realized the coprological and bile analysis; LC performed the histological analysis and analyzed the data. LC drafted the man­uscript. All authors read and approved the final manuscript.
